# Getting smart to cognitive enhancers

**DOI:** 10.1016/j.eclinm.2019.06.014

**Published:** 2019-06-27

**Figure:**
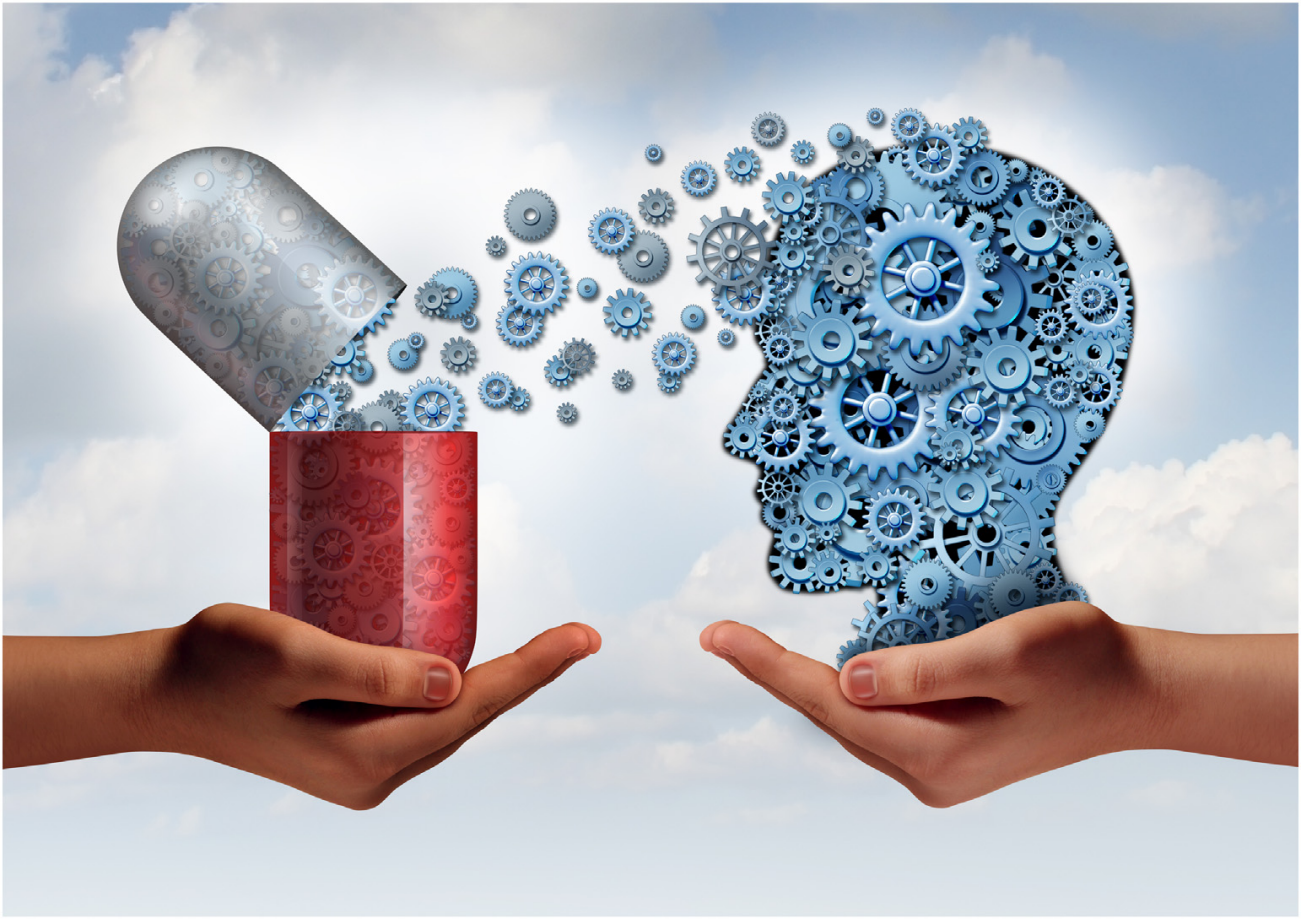

Curiosity and desire to explore the unknown are integral to human nature. They pertain to both the physical and cerebral world: people have built ships to discover new lands and have found ways to sail across non-ordinary states of consciousness. Evidence of the use of state-altering substances such as alcohol or caffeine dates back thousands of years; however, the recent finding of Melanie Miller at the University of Otago (Dunedin, New Zealand) and José Capriles at Pennsylvania State University (PA, USA), and their colleagues, published in *Proceedings of the National Academy of Sciences of the United States of America*, documents a rarer event. Hidden in the highland Andes, they found the resting place of an indigenous shaman who lived 1000 years ago, buried with a ritual bundle, which contained multiple psychoactive plants, including coca and hallucinogenic plants. This bundle, designed for the consumption of mind-altering drugs, is a sign not only of the intricate botanical knowledge required for pre-Columbian ritual practices, but also of the ancient and cross-cultural nature of the human will to push our brain beyond its limits.

Today, this same wish persists, and we can see its expression in many diverse ways. In science fiction, the idea of a future where we can rapidly acquire knowledge and skills, bypassing the usual demanding and time-consuming routes, is a common theme. In the film *The Matrix*, which was released 20 years ago, it is through augmentation of the mind, and not the physical body, that the characters achieve the most powerful abilities. Just a couple of years later, Alan Glynn’s book *The Dark Fields*, which inspired the film *Limitless*, presented another fascinating temptation: an experimental drug that allows full use of the brain, and unlocks the normally out-of-reach astonishing abilities of the mind. After almost 20 years, are we reaching a point where those dreams are an accessible reality?

Microdosing of illegal psychedelic drugs such as lysergic acid diethylamide (LSD), for example, is becoming part of a daily routine for some people, to increase creativity and productivity. In addition, prescription legal drugs, originally developed as treatments for disorders such as attention-deficit hyperactivity disorder (ADHD) or sleep disorders, are becoming increasingly popular among the healthy population to maintain or improve work performance. The Global Drug Survey shows that, across 15 countries, the non-ADHD population who used pharmacological cognitive enhancers increased by 180% in 2017, when compared with 2015.

Cognitive enhancers (also known as nootropics, smart drugs, or brain boosters) are consumed in an attempt to improve memory, increase concentration, boost energy levels and wakefulness, and ameliorate mood. It can be confusing that the information available online often presents prescription nootropics together with other substances such as herbal supplements, amino acids, and brain nutrients, because a lack of clarity and definitions can be harmful for a population who is unfamiliar with the differences. But this is not the only concern: the growing use of neuroenhancers is giving rise to new ethical issues. One could claim that using smart drugs is similar to taking a short-cut—it allows you to perform better without actually making the effort to improve yourself—and this is opening a whole new field for careful ethical discussion. However, we still have too little information to establish the net effects and the risks versus benefits. Therefore, caution is required when prescribing or using these substances, until their safety and effectiveness is established. Evidence of the real effects of such interventions on cognitive performance and long-term side-effects are needed.

Research on cognitive enhancers is growing, and an increasing body of preclinical and clinical work will help not only to dissect the effect of smart drugs on the healthy population, but also to test whether those interventions could be used to treat brain-related disorders. Cognitive enhancers are being tested in depression, schizophrenia, Alzheimer’s disease, in ageing, and even to counterbalance the effects of relapse in substance misuse and addiction. Although the majority of such studies are still at a preclinical stage, some applications are getting closer to the clinic. Paul Newhouse, director of the Vanderbilt Center for Cognitive Medicine (Nashville, TN, USA) is working on a first-in-human phase 1 trial in healthy volunteers to establish the safety and tolerability of the putative cognitive enhancer VU319. If the trial is successful, the drug could be then tested for its cognitive enhancement capacity in the context of Alzheimer's disease (NCT03220295). Discovering new molecules is not the only way to proceed. Repurposing of commercially available cognitive enhancers can expand their range of application, and David Devos from the University Hospital Center of Lille (Lille, France), is now recruiting patients to test whether a low dose of methylphenidate, a drug normally used to treat ADHD, could be used in slight to mild cognitive disorders in the elderly (NCT03280251). However, the use of nootropics therapeutically requires the most conscientious ethical considerations. Patients affected by dementia have had their expectations raised and dashed too many times, and future trials will need to have robust design and execution to avoid fostering false hope.

While the smart drugs field is exploding, thorough research to assess their safety and applicability in health and disease is needed. *EClinicalMedicine* is eager not only to promote such research, but also to encourage discussion on the topic, with the goal of tracing a path towards the safe and ethical use of nootropics.

*EClinicalMedicine*

